# Lipodystrophic syndromes due to *LMNA* mutations: recent developments on biomolecular aspects, pathophysiological hypotheses and therapeutic perspectives

**DOI:** 10.1080/19491034.2018.1456217

**Published:** 2018-04-16

**Authors:** Corinne Vigouroux, Anne-Claire Guénantin, Camille Vatier, Emilie Capel, Caroline Le Dour, Pauline Afonso, Guillaume Bidault, Véronique Béréziat, Olivier Lascols, Jacqueline Capeau, Nolwenn Briand, Isabelle Jéru

**Affiliations:** a Sorbonne Université, Inserm UMR_S 938, Centre de Recherche Saint-Antoine, Institut Hospitalo-Universitaire de Cardio-métabolisme et Nutrition (ICAN), Paris, France; b Assistance Publique-Hôpitaux de Paris, Hôpital Saint-Antoine, Laboratoire Commun de Biologie et Génétique Moléculaires, Paris, France; c Assistance Publique-Hôpitaux de Paris, Hôpital Saint-Antoine, Centre National de Référence des Pathologies Rares de l'Insulino-Sécrétion et de l'Insulino-Sensibilité (PRISIS), Service d'Endocrinologie, Diabétologie et Endocrinologie de la Reproduction, Paris, France; d Wellcome Trust Sanger Institute, Wellcome Trust Genome Campus, Hinxton, UK; e University of Cambridge Metabolic Research Laboratories, Wellcome Trust-MRC Institute of Metabolic Science, Addenbrooke's Hospital, Cambridge CB2 0QQ, UK; f Department of Molecular Medicine, Institute of Basic Medical Sciences, Faculty of Medicine, University of Oslo, Blindern, Oslo, Norway

**Keywords:** Lamin A/C, lipodystrophy, adipose tissue, differentiation, senescence, extracellular matrix, anticipation, epigenetics, induced pluripotent stem cells, metreleptin

## Abstract

Mutations in *LMNA*, encoding A-type lamins, are responsible for laminopathies including muscular dystrophies, lipodystrophies, and premature ageing syndromes. *LMNA* mutations have been shown to alter nuclear structure and stiffness, binding to partners at the nuclear envelope or within the nucleoplasm, gene expression and/or prelamin A maturation. *LMNA*-associated lipodystrophic features, combining generalized or partial fat atrophy and metabolic alterations associated with insulin resistance, could result from altered adipocyte differentiation or from altered fat structure.

Recent studies shed some light on how pathogenic A-type lamin variants could trigger lipodystrophy, metabolic complications, and precocious cardiovascular events. Alterations in adipose tissue extracellular matrix and TGF-beta signaling could initiate metabolic inflexibility. Premature senescence of vascular cells could contribute to cardiovascular complications. In affected families, metabolic alterations occur at an earlier age across generations, which could result from epigenetic deregulation induced by *LMNA* mutations. Novel cellular models recapitulating adipogenic developmental pathways provide scalable tools for disease modeling and therapeutic screening.

## Introduction

Lipodystrophic syndromes are rare diseases characterized by generalized or partial fat atrophy (lipoatrophy) and metabolic alterations resulting from insulin resistance (glucose tolerance abnormalities, dyslipidemia, non-alcoholic fatty liver disease). Obesity and lipodystrophy share similar metabolic defects thus illustrating the complex relationships between deregulation of adipose tissue and systemic metabolism. Several authors postulate that a personal threshold controls the individual capacity to store nutrient excess as triglycerides in the lipid droplet of white subcutaneous adipocytes [1–3] When the nutrient intake exceeds adipose tissue storage capacity, this results in ectopic lipid deposition in non-adipose tissues and in metabolic inflexibility. These alterations characterize both metabolically-unhealthy obesity and lipodystrophic diseases. Accordingly, a recent genomic study, performed in the general population, has revealed that limited peripheral adipose storage capacity is a major determinant of insulin resistance [4].

Important questions remain unanswered regarding factors that modulate the personal threshold of adipocyte expandability. These factors depend on multiple mechanisms, notably formation of new adipocytes from precursors (adipogenesis) and biogenesis, maintenance and regulation of the adipocyte lipid droplet, but also interactions between adipocytes and other cellular and extracellular components of adipose tissue, and cross-talks between the different body fat depots and other organs.

**Figure 1.**
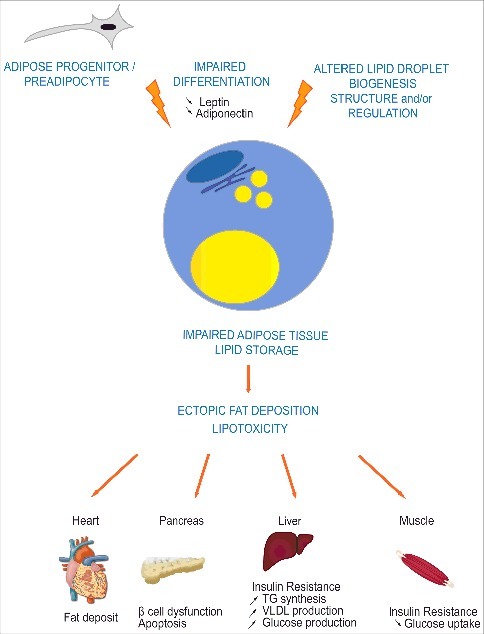
Impaired adipose tissue lipid storage induces metabolic complications in lipodystrophic syndromes. Most genes involved in lipodystrophic syndromes have been shown to regulate adipocyte differentiation, triglycerides synthesis, lipolysis, and/or lipid droplet structure or biogenesis. Impaired storage of excess energy as triglycerides in adipocytes leads to ectopic fat deposition and lipotoxicity in several tissues such as muscle, heart, liver and pancreas, resulting in post-receptor insulin resistance, dyslipidemia and liver steatosis.

Although lipodystrophic syndromes are rare diseases, deciphering their pathogenic mechanisms would provide valuable insights into adipose tissue physiology, notably regarding its capacity to adequately expand and maintain metabolic homeostasis. Major advances have been achieved since 1999 thanks to the identification of several monogenic causes of lipodystrophic syndromes [5–18]. Their description is far beyond the scope of this review, but it is worth mentioning that pathophysiological molecular mechanisms of most of them involve defects in adipocyte differentiation, in biogenesis and/or structural properties of the adipocyte lipid droplet, in triglyceride synthesis and/or in lipolysis [19]. This further stresses the important role played by the lack of lipid storage in the occurrence of post-receptor insulin resistance (Figure 1) [20]. *LMNA* mutations are responsible for the most frequent genetic form of lipodystrophy. However, the mechanisms by which these molecular variants alter adipocyte function remain largely unknown. Strikingly, the different *LMNA* mutations, located all along the gene, give rise to very diverse clinical phenotypes of laminopathies, comprising not only lipodystrophic syndromes but also dystrophic myopathies, neuropathies, premature ageing syndromes and rare overlapping syndromes. This very large clinical spectrum associated with *LMNA* defects, illustrates the pathophysiological complexity of laminopathies [21].

After a brief overview of the pathogenic mechanisms that have been discussed since the discovery of the first laminopathies in 1999 [22], we will propose an update of some recent studies on *LMNA*-related lipodystrophies, dealing with some genetic, preclinical and fundamental aspects, which allow to refine or readdress the pathophysiological hypotheses.

## Several pathogenic mechanisms contribute to laminopathies

A- and B-type lamins are nuclear proteins belonging to the intermediate filaments family. Lamins have been shown to play an essential role in nuclear function.

Lamins A and C, both encoded by *LMNA*, are developmentally regulated in most lineage precursors and are expressed in differentiated cells. They represent A-type lamins main isoforms and interact with the ubiquitous B-type lamins, encoded by *LMNB1* and *LMNB2*. The lamin functional domains are organized into a short N-terminal head domain, a central alpha helical rod domain, driving lamin dimerization and polymerization, and a large C-terminal tail, containing the nuclear localization signal and an immunoglobulin-like fold domain with multiple binding properties [23]. A CaaX motif, at the C-terminal end, allows post-translational farnesylation of both prelamin A isoform produced by *LMNA* and B-type lamins. While B-type lamins retain the farnesyl moiety, thus increasing their affinity for the inner nuclear membrane, prelamin A undergoes further post-translational modifications. Farnesylated prelamin A is finally cleaved by the ZMPSTE24/FACE-1 metalloproteinase, removing its farnesylated C-terminal end, and producing a mature, non-farnesylated lamin A [24].

Lamin filaments form the lamina meshwork at the nucleoplasmic side of the inner nuclear membrane, which provides a structural support for the nucleus [25,26], and controls the functional organization of interphase chromatin [27]. At the inner nuclear periphery, lamins interact with several inner nuclear membrane proteins. Among them, the SUN-domain proteins span the inner nuclear membrane and bind to the KASH domain of proteins embedded in the outer nuclear membrane, which, in turn, bind to cytoskeletal proteins. All these proteins together form a complex that links the nucleoskeleton to the cytoskeleton [28–30]. Lamin-associated nuclear envelope proteins can impact on chromatin, and influence the spatial positioning of developmental genes in a tissue-specific manner [31,32]. Through these multistep interactions, lamins control nuclear stiffness and mechano-sensitivity, which are strongly modified during stem cell differentiation [33–35]. In addition, A- type lamin filaments, although mainly localized at the nuclear periphery, are also found in the nucleoplasm, where they interact with lamina-associated protein 2alpha (LAP2alpha), a modulator of cell-cycle progression and apoptosis [36], and where they regulate several other signaling proteins and transcription factors [37]. Lamins also bind DNA and histones, ensuring the formation of multiprotein complexes associated with chromatin, able to regulate the expression of genes such as retinoblastoma protein (Rb) and barrier-to-integration factor (BAF) [37]. Importantly, lamins organize chromatin at the nuclear periphery through lamin-associated domains (LAD) [38], and regulate interactions with epigenetic factors such as the Polycomb group of proteins [39]. Thus, there is increasing evidence that A-type lamins epigenetically influence stem cell differentiation and tissue-specific developmental programs [40–42]. As many structural and regulatory roles of A-type lamins are impaired by *LMNA* mutations, the pathophysiological mechanisms of the different laminopathies could involve distinct pathways.

## Defects in adipocyte differentiation in *LMNA*-associated lipodystrophies

The clinical features of *LMNA*-associated lipodystrophic syndromes are reviewed by David Araujo-Vilar in this journal issue. The typical familial partial lipodystrophy of the Dunnigan type (FPLD2, OMIM #151660) is mainly due to heterozygous amino acid substitutions at the 482nd position in the C-terminal domain of A-type lamins, the most frequent being the p.Arg482Trp variant. Closely related lipodystrophic phenotypes are due to other point mutations in the immunoglobulin-fold domain [6,7,43]. In contrast to *LMNA* mutations involved in muscular dystrophies or cardiomyopathies, lipodystrophy-causing mutations do not disrupt the tridimensional structure of A-type lamins but modify a positively charged amino acid at the surface of their C-terminal domain [44,45].

In accordance, several studies have confirmed that *LMNA* mutations specific for lipodystrophies result in modified interactions of the protein C-terminal domain with distinctive partners *in vitro*. Thus, *in vitro* studies have revealed that two FPLD-causing mutations, *LMNA* p.Gly465Asp and p.Lys486Asn, alter the lamin A C-terminal tail SUMOylation, a posttranslational modification known to regulate the localization, interactions and functions of proteins [46]. The *LMNA* p.Arg482Leu mutation down-regulates Notch signaling in mesenchymal stem cells, decreasing their adipogenic potential [47]. SREBP1c, an important transcription factor driving adipogenesis, binds differently wild-type and lipodystrophy-causing lamin A variants [48,49]. In addition, the *LMNA* p.Arg482Trp and p.Arg482Gln mutations impair the interaction between lamin A and DNA *in vitro* [50]. It has been shown that lamin A, SREBP1 and its DNA responsive elements form ternary complexes *in vitro*, and that Arg482Trp lamin A deregulates SREBP1 activity in patients’ cells [51], suggesting that it could disrupt adipocyte differentiation. The expression of Arg482Trp or Arg482Gln lamin A, but also overexpression of wild-type lamin A, inhibit adipocyte differentiation of 3T3-L1 cells [52]. Recently, the Fragile X related protein (FXR1P), a mRNA binding protein, was identified as a lamin A partner at the nuclear envelope. Its expression and localization inside the nucleus are modified in the presence of lamin A bearing lipodystrophy-causing mutations [53]. The expression of Arg482Trp lamin A in human adipose stem cells increases FXR1P protein expression and impairs adipocyte differentiation through a process involving epigenetic and conformational changes in chromatin organization [42,53].

At the clinical level, FPLD2 illustrates how the different body fat depots, which are characterized by distinct developmental origins [54], respond in a very different manner to the same constitutional *LMNA* mutation. Indeed, while patients’ subcutaneous fat mass at the limbs and buttocks level is severely decreased, the mass of cervical, facial, perineal and visceral depots is increased. In addition, the lipodystrophic phenotype becomes apparent generally after puberty, and is more pronounced in women [43,55,56]. In agreement with the hypothesis of impaired adipogenesis induced by *LMNA* mutations, we and others reported that expression of adipogenic genes was altered in adipose tissue from patients with FPLD2, both at thigh [57,58] and cervical levels [59], with a decreased expression of the master adipogenic factor PPAR-gamma. Dystrophic features characterized not only lipoatrophic adipose tissue, but also lipomatous areas, and accumulated cervical fat, from patients with FPLD2 [57–59].

In addition to FPLD2, due to hotspot mutations in the C-terminal region, lipodystrophic features are also observed in uncommon forms of complex laminopathies due to mutations affecting different protein domains of A type-lamins. These mixed forms associate lipodystrophy and muscular and/or cardiac symptoms [60–62], and also often signs of premature aging [63–69]. Mandibulo-acral dysplasia, due to mutations in *LMNA* or *ZMPSTE24*, was identified as the first laminopathy associating premature ageing and lipodystrophic features [63,64]. This phenotypic combination was further observed with other *LMNA* mutations in typical Hutchinson-Gilford progeria [66,67] or in atypical progeroid syndromes [65,68,69].

In that setting, similar cellular defects have been observed in different laminopathies. Nuclear abnormal morphology and nuclear envelope disorganization appear as hallmarks of human cultured laminopathic cells, independently of the associated clinical presentation [66,67,70–75]. The typical *LMNA* mutation responsible for Hutchinson-Gilford progeria results in the expression of a constitutively farnesylated prelamin A pathogenic variant, called progerin. Although other lipodystrophy-causing mutations in *LMNA* do not directly modify the proteolytic maturation site of the protein, they could secondarily alter its maturation and result in prelamin A accumulation. Accordingly, we and others observed an accumulation of prelamin A in cells and/or tissues from patients with FPLD2 or other *LMNA*-related lipodystrophies associated or not with progeroid signs [57,76,77]. Although this prelamin A accumulation is controversial [78], some HIV protease inhibitors, used as antiretroviral drugs, and involved in the development of a lipodystrophic syndrome with premature cellular senescence [75], were also shown to increase the cellular amount of farnesylated prelamin A through inhibition of ZMPSTE24 [77,79]. Prelamin A accumulation, by sequestrating SREBP1c at the nuclear periphery, may alter adipogenesis [76,80,81]. Accumulated prelamin A could sequestrate the transcription factor Sp1, resulting in altered extracellular matrix gene expression and adipose lineage differentiation of human mesenchymal stem cells [82]. In addition, prelamin A and progerin were shown to induce the recruitment of the chromatin remodeling factor BAF inside the nucleus, which could result in altered gene expression [83,84].

However, accumulation of farnesylated prelamin A is not mandatory for the development of lipodystrophic diseases upon expression of lipodystrophy-causing lamin A variant. We have described a pathogenic homozygous frameshift mutation in *LMNA*, leading to the synthesis of a prelamin A variant lacking the consensus CaaX farnesylation site. This variant results in the expression of a non-farnesylated form of prelamin A, without any production of mature lamin A, and is responsible for a severe lipodystrophic syndrome [85]. Other studies also showed that observation of prelamin A accumulation may depend on the antibodies used, and is not a prerequisite for lipodystrophy diseases [78,86].

Taken as a whole these studies show that lipodystrophy-causing *LMNA* mutations could result in several defects leading to defective adipocyte differentiation. Several recent studies extend and refine these hypotheses, further linking pathogenic molecular mechanisms to clinical features.

## Early extracellular matrix alterations in lipodystrophic laminopathies

Altered adipose tissue extracellular matrix (ECM), acknowledged as a major contributor to metabolic alterations associated with obesity [87], has also been observed in patients with lipodystrophic syndromes of different etiologies [15,17,59,88].

Le Dour et al generated transgenic mice overexpressing the human p.Arg482Gln pathogenic lamin A variant specifically in adipose tissue, and also studied a transgenic mice expressing higher levels of p.Arg482Gln lamin A [89]. The severity of the lipodystrophic phenotype in mice probably depends on the level of expression of p.Arg482Gln lamin A, since only the latter mouse model displayed a decreased capacity to accumulate body fat, associated with decreased insulin sensitivity and liver steatosis [90]. However, ECM alterations were observed in adipose tissue from mice overexpressing p.Arg482Gln lamin A only in fat tissue, similar to those reported in adipose tissue from patients with FPLD2, even though these mice did not show overt lipoatrophy. This suggests that these tissular abnormalities may be early defects in the pathogenesis of the disease [59,90]. Indeed, human and mice subcutaneous adipose tissue expressing p.Arg482Gln lamin A displayed increased fibrosis and decreased mean adipocyte area. In addition, the level of gene expression of fibronectin, which binds type 1 collagen and is involved in the maintenance of adipocyte shape, was increased. Conversely, the level of gene expression of elastin, a major component of elastic fibers providing strength and flexibility to connective tissue, and of decorin, which also binds to type 1 collagen and participates to matrix assembly, was decreased. Similar ECM abnormalities were also observed in cultured fibroblasts from patients with FPLD2 or other *LMNA*-associated lipodystrophic syndromes. These abnormalities were linked to activation of TGF-beta signaling, a driver of matrix deposition, and were associated with increased expression and activity of matrix metalloproteinase 9, an endopeptidase that degrades ECM proteins.

**Figure 2.**
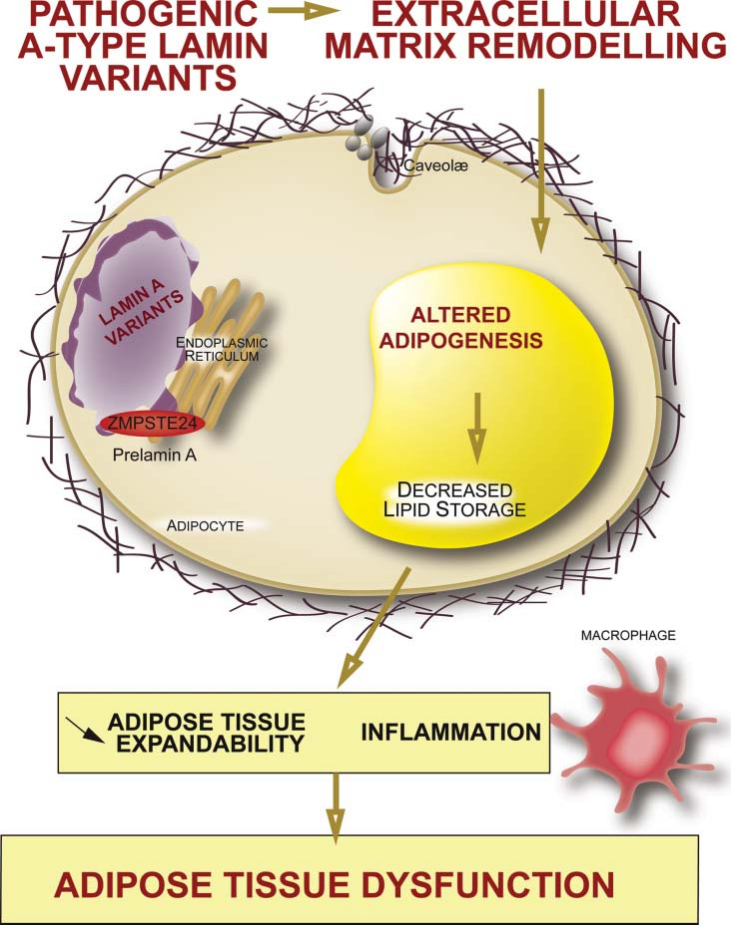
An early detrimental remodeling of adipose tissue extracellular matrix could contribute to the pathophysiology of *LMNA*-associated lipodystrophies. Extracellular matrix alterations induced by lipodystrophy-causing *LMNA* mutations could hamper adipocyte differentiation and limit the expandability of adipose tissue, triggering adipocyte dysfunction and metabolic defects.

These results suggest that an early detrimental remodeling of fat ECM develops upon expression of lipodystrophy-causing lamin A variants in adipose tissue. This could hamper adipocyte differentiation and contribute to the limited capacity of fat storage, previously shown to induce adipose tissue dysfunction, fatty acid spillover to non-adipose organs, lipotoxicity and associated metabolic defects in humans (Figure 2).

**Figure 3.**
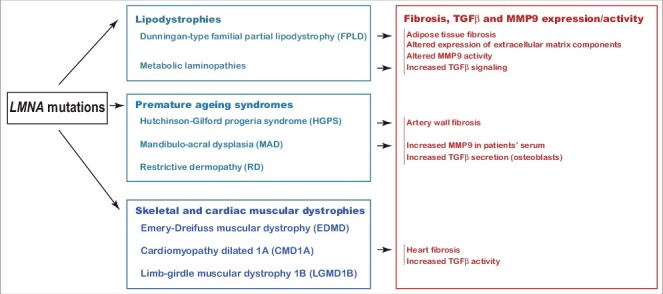
Several laminopathies are characterized by tissular fibrosis, increased TGF-beta signaling and/or metalloproteinase expression/activity. Extracellular matrix alterations at the level of adipose tissue, vascular wall or heart have been described in several laminopathies and participate to the clinical phenotype and to the complications of laminopathies. ^59^^,^^90-96^

ECM alterations with fibrosis, altered metalloproteinase activity and/or increased TGF-beta signaling have been previously described in *LMNA*-linked cardiomyopathy [91,92], mandibulo-acral dysplasia [93–95], and Hutchinson-Gilford progeria (Figure 3) [96]. This suggests that ECM alterations, triggered by several *LMNA* mutations in different tissues, could globally contribute to the pathophysiology of laminopathies, and that antagonists of TGF-beta may have potential therapeutic benefit in these diseases.

## Premature senescence and osteoblast-like differentiation of smooth vascular cells in lipodystrophic laminopathies

Cardiovascular events are frequent and precocious in patients with FPLD2, inasmuch as they are frequently exposed to dyslipidemia, insulin resistance and/or diabetes [97,98]. However, in addition to metabolic risk factors, FPLD2-associated *LMNA* mutations could have a direct impact on the vascular wall cells.

**Figure 4.**
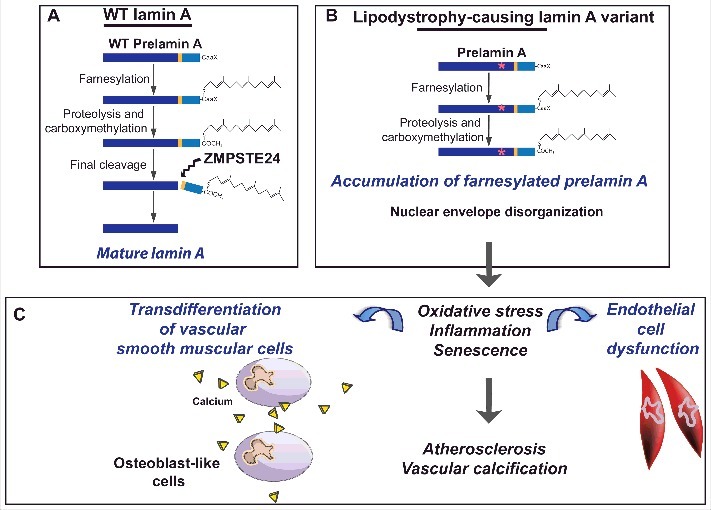
Vascular effects of *LMNA* mutations causing lipodystrophy. A. Prelamin-A physiologically undergoes a complex post-translational maturation process affecting its C-terminal CaaX motif. After farnesylation of the carboxy-terminal cysteine, aaX amino acids are removed, farnesyl-cysteine is carboxymethylated, then the 15 C-terminal amino acids are cleaved by the metalloprotease ZMPSTE24 to produce mature lamin A. B. Lipodystrophy-causing mutations in *LMNA* lead to accumulation of farnesylated prelamin A and nuclear envelope disorganization. C. Accumulation of prelamin A pathogenic variants at the nuclear envelope induces oxidative stress, inflammation and cellular senescence. These cellular alterations contribute to endothelial cell dysfunction and to osteoblastic transdifferentiation of vascular smooth muscle cell, promoting atherosclerosis and vascular calcification.

In Hutchinson-Gilford progeria, severe premature atherosclerosis leads to myocardial infarction and strokes, the major causes of patients’ death at a mean age of 14.6 years. This has been linked to accumulation of progerin, a farnesylated mutated form of prelamin A expressed in patient's cells [99–101]. We observed, in FPLD2, that p.Arg482Trp prelamin A accumulated abnormally at the nuclear envelope and induced endothelial cell dysfunction with increased oxidative stress and cellular senescence [98]. Additionally, we recently showed that several *LMNA* mutations, either leading to a lipodystrophy typical of the FPLD2 type, or associated with signs of premature ageing, also triggered vascular smooth muscle cell senescence with osteoblastic transdifferentiation and calcification [102]. This could lead to early vascular calcifications, as observed in patients [102]. All together, these studies suggest that *LMNA* mutations responsible for lipodystrophies may directly affect the arterial wall, resulting in early atherosclerosis and vascular calcification, in addition to atherosclerotic lesions resulting from associated metabolic risk factors (Figure 4). In human induced pluripotent stem cells, p.R482W lamin A was recently shown to deregulate the network of genes involved in early vascular differentiation, which is also in favor of a cell-autonomous origin of endothelial cell dysfunction in FPLD2 [103]. This should encourage researchers to develop therapeutic strategies aiming at minimizing the cellular amount and toxicity of pathogenic A-type lamin variants, not only in Hutchinson-Gilford progeria but also in *LMNA*-linked lipodystrophic diseases.

## Anticipation of metabolic complications in lipodystrophic laminopathies

**Figure 5.**
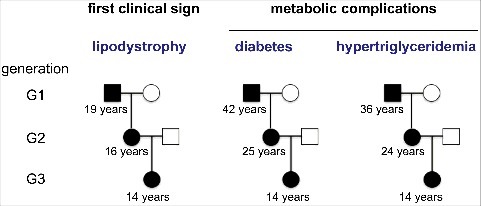
Anticipation of metabolic complications in lipodystrophic laminopathies. The comparison of the natural history and disease severity in familial forms of lipodystrophic syndromes due to *LMNA* pathogenic variants reveals similar characteristics and age at onset for lipodystrophy, but an anticipation of metabolic complications over generations. The mean age at onset of each clinical sign is indicated below symbols representing patients from different generations.

The study of our cohort, that represents the largest cohort of familial forms of *LMNA*-associated lipodystrophies reported to date (85 patients from 24 families), revealed that diabetes and hypertriglyceridemia occurred at an earlier age over successive generations (Figure 5) [104]. This happened independently of the potential earlier screening of metabolic alterations over time. In contrast, lipodystrophy, which is the earliest clinical feature, appeared at similar age in all patients. Notably, body mass index and total fat mass were similar in patients from different generations, showing that these factors cannot account by themselves for this observation. This major decrease in the age at onset of metabolic complications provides one of the very rare examples of anticipation in a Mendelian disorder that does not fit with the well-known model of trinucleotide repeat disorders [105]. Recognition of this phenomenon is important for proper management of the disease. In this regard, we propose to perform presymptomatic genetic screening during childhood in affected families, with reinforcement of metabolic monitoring and adoption of preventive lifestyle measures in young affected patients.

This anticipation phenomenon has to be considered in the light of recent studies, showing that *LMNA* mutations could modify, by epigenetic mechanisms, the chromatin architecture regulated by lamins during development [38,106–108]. Complications of lipodystrophic laminopathies might thus be sensitive to environmental agents triggering chromatin rearrangements. As an example, several environmental stressors, including prenatal exposure to parental disease, have been shown to drive type 2 diabetes inheritance at the epigenetic level [109]. Disruptions in gene expression could alter either adipocyte [42,110,111] or myogenic [41] cell differentiation, depending on the type of *LMNA* mutation. These studies, reviewed by Briand and Collas in this issue, suggest that lamin A mutations could differently alter the cell fate of different cell lineages. They provide major keys to understand how different pathogenic mutations in the same *LMNA* gene can lead to tissue-specific phenotypes in humans.

## Recombinant leptin therapy in the management of metabolic alterations in patients with *LMNA*-associated lipodystrophies

International guidelines for the management of lipodystrophy syndromes were recently published [112]. Patients with *LMNA*-associated lipodystrophies are mainly treated with therapies recommended for classical diabetes and dyslipidemia. Among them, diet and exercise are of major importance to reduce insulin resistance and metabolic complications. Metformin is a first-line therapy for insulin resistance and diabetes. Hypoglycemic agents, including insulin, can be useful, although their efficiency has not been specifically studied in these rare diseases. Lipids should also be managed in accordance with guidelines for the general population, although stricter targets for LDL-cholesterol may be discussed in the presence of several metabolic and cardiovascular risk factors. There is no current treatment that can reconstitute adipose tissue, but plastic surgery can be helpful when lipodystrophy causes psychological distress and/or physical discomfort [112].

Leptin deficiency, which correlates with the decreased amount of body fat, was shown to contribute to metabolic complications of lipodystrophies, whatever their underlying molecular mechanisms, both in mice [113,114] and humans [115,116]. Recombinant leptin (metreleptin) therapy is approved in the US and Japan for the treatment of lipodystrophic syndromes, and is available through named compassionate programs in several European countries. Treatment with metreleptin decreases insulin resistance, hyperglycemia, dyslipidemia and liver steatosis in hypoleptinemic lipodystrophic patients, in part independently of an improved control of deregulated eating behavior [115–118]. However, this treatment is less efficient in partial forms of lipodystrophies than in generalized ones [119]. We have recently shown that metreleptin improves not only insulin sensitivity, but also insulin secretion in patients with lipodystrophies, which could result from decreased lipotoxicity in pancreatic islets [120]. Insulin secretion also improved under metreleptin therapy in the subgroup of patients with lipodystrophic syndromes due to *LMNA* mutations, either of the FPLD2 type or associated with mixed laminopathic phenotypes. We also confirmed that the effect of metreleptin on glucose control was related to the severity of baseline hyperglycemia. In addition, we observed, in these patients, that one-year metreleptin therapy significantly decreased the plasma concentrations of proprotein convertase subtilisin/kexin type 9 (PCSK9) [121]. PCSK9 is an endogenous inhibitor of LDL receptor that increases LDL-cholesterol circulating levels. In accordance, metreleptin-mediated decrease in PCSK9 was associated with a reduction in the level of the proatherogenic apolipoprotein B [121].

These results further stress that metreleptin improves the metabolic consequences of *LMNA*-associated lipodystrophies and should be integrated in the therapeutic strategy [112].

## New cellular tools to study adipocyte development *in vitro*

As stated above, it is likely that developmental defects affecting the adipocyte lineage underlie, at least in part, important pathophysiological mechanisms leading to lipodystrophic laminopathies.

In that setting, reprogramming of patients’ primary cells into human induced pluripotent stem cells (hiPSCs) provides a relevant cellular model for pathophysiological studies. One limitation of this strategy is the incomplete knowledge of developmental pathways leading towards the distinct human adipose depots. Indeed, several types of adipocytes co-exist in the human body. Whereas adipocytes store excess energy as triglycerides, brown adipocytes are able to dissipate energy through mitochondrial thermogenesis [122]. A third type of adipocytes, called beige adipocytes, displays thermogenic properties upon activation, and could therefore be a relevant target to treat metabolic complications of diabetes [123].

Most of the current protocols for *in vitro* adipocyte differentiation of hiPSCs are based on generation of embryoid bodies and/or derivation of mesenchymal stem cells prior to adipose differentiation [124]. A strategy involving overexpression of adipogenic transcription factors has also been proposed [125], but this results in a bypass of developmental pathways and therefore hampers pathophysiological studies.

Guénantin, Briand et al have recently set up an efficient protocol allowing the differentiation of hiPSCs into adipose progenitors with a dual white and beige differentiation potential [126]. This protocol recapitulates adipocyte developmental processes *in vitro* through mesodermal then adipose stem cells stages. Engraftment of hiPSC-derived progenitor cells allowed the generation of a well-organized human adipose tissue *in vivo*.

**Figure 6.**
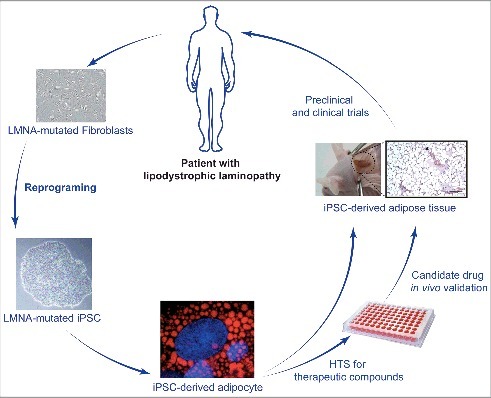
A new cellular tool for studying adipocyte development *in vitro.* Patients’ primary cells reprogramming into induced Pluripotent Stem Cells (iPSCs) allow to study several relevant cells types that would be otherwise inaccessible. This also avoids using non-physiological lamin overexpression strategies. Differentiation of iPS cells originating from patients with lipodystrophic laminopathies into adipocytes through a developmentally relevant protocol also provides an unlimited source of cells for high throughput screening (HTS) of therapeutic compounds, opening perspectives for the treatment of these rare diseases.

This new iPS cell-based tool may be particular relevant to study adipose differentiation in lipodystrophic laminopathies. In addition, this unlimited source of adipocytes could provide a valuable material for drug screening and further development of targeted therapeutic approaches (Figure 6).
